# Suppressor of Cytokine Signaling 1 Counteracts Rhesus Macaque TRIM5α-Induced Inhibition of Human Immunodeficiency Virus Type-1 Production

**DOI:** 10.1371/journal.pone.0109640

**Published:** 2014-10-13

**Authors:** Sayaka Sukegawa, Ryuta Sakuma, Seiga Ohmine, Hiroaki Takeuchi, Yasuhiro Ikeda, Shoji Yamaoka

**Affiliations:** 1 Department of Molecular Virology, Tokyo Medical and Dental University, Tokyo, Japan; 2 Department of Molecular Medicine, Mayo Clinic, Rochester, Minnesota, United States of America; University of Colorado Denver, United States of America

## Abstract

Old world monkey TRIM5α is a host factor that restricts human immunodeficiency virus type-1 (HIV-1) infection. Previously, we reported that rhesus macaque TRIM5α (RhTRIM5α) restricts HIV-1 production by inducing degradation of precursor Gag. Since suppressor of cytokine signaling 1 (SOCS1) is known to enhance HIV-1 production by rescuing Gag from lysosomal degradation, we examined if SOCS1 is involved in RhTRIM5α-mediated late restriction. Over-expression of SOCS1 restored HIV-1 production in the presence of RhTRIM5α to a level comparable to that in the absence of RhTRIM5α in terms of titer and viral protein expression. Co-immunoprecipitation studies revealed that SOCS1 physically interacted with RhTRIM5α. Over-expression of SOCS1 affected RhTRIM5α expression in a dose-dependent manner, which was not reversed by proteasome inhibitors. In addition, SOCS1 and RhTRIM5α were detected in virus-like particles. These results suggest that SOCS1 alleviates RhTRIM5α-mediated regulation in the late phase of HIV-1 life cycle probably due to the destabilization of RhTRIM5α.

## Introduction

Old world monkey TRIM5α was originally identified as an intrinsic immune agent that blocks human immunodeficiency virus type-1 (HIV-1) infection immediately after viral entry [1]. TRIM5α carries RING, B-box2, coiled-coil (RBCC) and B30.2/SPRY domains. In the post-entry restriction, RhTRIM5α recognizes incoming viral cores, but not the capsid protein as a monomer, through the B30.2 domain. The B30.2 domain determines the antiviral spectrum and magnitude of post-entry restriction. The B-box2 and the coiled-coil domains are required to form homo/hetro-multimer [2]–[4] and the B30.2 domains of multimerized TRIM5α stick in the grooves on the surface of incoming viral cores [5], [6]. After recognizing the structured core, RhTRIM5α induces aberrant disassembly of core, resulting in the disruption of reverse-transcription of viral genomic RNA [1].

We previously reported that RhTRIM5α also restricts HIV-1 production by a mechanism distinct from that of its post-entry restriction [7]; RhTRIM5α targets precursor Gag (pr55^Gag^) to induce its degradation in a proteasome-independent manner. RhTRIM5α-mediated late restriction is a cell-line specific event; HEK293T cells support its antiviral activity, yet TE671 cells do not [8], [9]. RhTRIM5α can be incorporated into virus-like particles (VLPs) made with codon-optimized Gag [10]. This suggested physical interaction between RhTRIM5α and pr55^Gag^, yet no direct evidence for it has been obtained. The RBCC domain defines the specificity of restriction; a human TRIM5α mutant carrying part of the B-box2 and coiled-coil domains of RhTRIM5α can block HIV-1 production. Mutations in the coiled-coil domain of RhTRIM5α inhibit Gag degradation, but not VLP-incorporation [10].

Suppressor of cytokine signaling 1 (SOCS1) is a negative regulator for innate and adaptive immunities [11]–[13]. Its expression is induced by interferon stimulation and suppresses cellular signals stimulated by cytokines such as type I interferon through the inhibition of STAT phosphorylation [14]. SOCS1 has an E3 ubiquitin ligase activity [15], [16]. Several recent reports strongly suggested that HIV-1 controls SOCS1 expression to replicate efficiently *in vivo* [17]–[22]. Yadav A et al. reported that the level of SOCS1 mRNA is elevated in CD4+ T cells isolated from HIV-1 carriers in comparison to healthy donors and natural HIV-1 suppressors [22]. Additionally, it was reported that SOCS1 increases HIV-1 production by rescuing pr55^Gag^ from its lysosomal degradation [23], [24]. SOCS1 directly interacts with the matrix and nucleocapsid regions of pr55^Gag^ and enhances its stability, resulting in the efficient production of progeny virions [24]. Those observations prompted us to examine if SOCS1 influences RhTRIM5α-mediated restriction of HIV-1 production. In the present study, we show that SOCS1 counteracts RhTRIM5α-mediated restriction of HIV-1 production.

## Materials and Methods

### Cells

HEK293T, TE671 [25] and TZM-bl [26]–[30] cells were maintained in Dulbecco's Modified Eagle's Medium with 4.5 g/L glucose (Sigma-Aldrich, St. Lois, MO), supplemented with 10% fetal bovine serum, 100 U/ml penicillin and 100 mg/ml streptomycin in a 37°C and 5.0% CO_2_ environment.

### Plasmids

RhTRIM5α expression plasmids with an HA tag at its C-terminus, pRhTRIM5α-HA was described previously [7]. Proviral plasmid pNL4-3 cloned in pUC18 was obtained from the NIH AIDS Research and Reference Reagent Program [31]. To prepare a human SOCS1 (HuSOCS1) expression construct, SOCS1 cDNA was amplified by reverse-transcription and polymerase chain reaction (PCR) using total RNA from HEK293T cells as the template. Total RNA was obtained from HEK293T cells with Tri-reagent (Molecular research center, Cincinnati, OH) and subjected to reverse-transcription by Superscript III cDNA synthesis kit (Thermo fisher scientific, Waltham, MA) with oligo(dT)_20_ primer. SOCS1 cDNAs were amplified with specific primers (5′-GCGAATTCCCACCATGGTAGCACACAACC-3′ and 5′-CGCTCGAGAATCTGGAAGGGGAAGG-3′). The amplified fragment was cloned into the EcoRI and XhoI sites of pcDNA3.1 (Thermo fisher scientific), and then the sequence of SOCS1 was validated by the BigDye 3.1 sequencing method (Thermo fisher scientific).

### Virus production

HEK293T cells (3.0×10^5^ cells/well in a 12-well plate) were co-transfected with 0.1 µg of pNL4-3, 0.3 µg of pRhTRIM5α-HA and 0.6 µg of pHuSOCS1 using FuGENE6 (Promega, Madison, WI) according to the manufacturer's instructions. The total amount of plasmids transfected was adjusted to 1.0 µg per sample with pcDNA3.1. For experiments with increasing amounts of pHuSOCS1 in the presence or absence of HIV-1, HEK293T cells were transfected with 0.1 µg of pUC18 or pNL4-3, 0, 0.0375, 0.075, 0.15, 0.3 or 0.6 µg of pHuSOCS1 and with or without 0.3 µg of pRhTRIM5α-HA. The total amount of plasmids was adjusted to 1.0 µg per sample with pcDNA3.1. To block proteasome-dependent protein degradation, cells were treated with 30 µM of MG115 (Sigma-Aldrich) or 30 µM MG132 (Sigma-Aldrich) for 16 hours.

### Virus titration

Two days after transfection, supernatants of transfected cells were harvested through a 0.45 µm syringe filter (EDM Millipore, Billerica, MA). TZM-bl indicator cells (1.25×10^4^ cells/well in a 48-well plate) were infected with 100 µl of viral supernatant. Forty-eight hours post-infection, infected cells were lysed with 100 µl of lysis buffer (25 mM Tris-HCl pH 7.8, 8.0 mM MgCl_2_, 1.0 mM DTT, 1.0% TritonX-100, 15% Glycerol) and luciferase activity in the lysate was determined. Additionally, the amount of p24 antigen in the supernatants was determined by p24-specific enzyme-linked immunosorbent assay (ELISA) kit (Zeptmetrix, Buffalo, NY) according to the manufacturer's instructions.

### Immunoblot analysis

Two days after transfection, whole cell lysates were harvested in radio immuno-precipitation assay (RIPA) buffer (50 mM Tris pH 7.2, 1.0% NP-40, 2.5 mg/ml sodium-deoxycholate, 9.0 mg/ml NaCl, 0.1% SDS, 1.0 mM EDTA). The concentration of protein was assayed by DC protein assay (Bio-rad laboratories, Hercules CA) according to the manufacturer's instructions. Then, 24 µg of cell lysates were separated in 10% polyacrylamide gels. Proteins transferred onto a PVDF membrane were detected by rat anti-HA (clone 3F10, Roche applied science, Mannheim Germany), rabbit anti-SOCS1 (clone A156, Cell signaling technology, Danvers, MA), mouse anti-HIV-1 p24 (clone 39/5.4A, Abcom, Cambridge, MA), human anti-IκBα (#sc-371, Santa cruz biotechnology, Dallas, TX) or mouse anti-GAPDH (clone 3H12, Medical and biological laboratories, Aichi, Japan) antibodies and visualized by horseradish peroxidase-conjugated goat anti-rat IgG (#sc-2006, Santa cruz biotechnology), goat anti-rabbit IgG (#7074, Cell signaling technology) or goat anti-mouse IgG (#32430, Thermo fisher scientific). For multiple probing, membranes were treated with the stripping buffer (2.0% SDS, 62.5 mM Tris-HCl pH6.8, 0.5% 2-mercaptoethanol) at 50°C for more than an hour, blocked again and then subjected to reaction with another primary antibody.

### Knockdown of SOCS1

Six siRNAs targeting SOCS1 were employed; 5′-GGCCAGAACCTTCCTCCTC-3′ [24] and 5′-CTGGGATGCCGTGTTATTT-3′ [19] and stealth siRNAs (5′-ACACAACCAGGTGGCAGCCGACAAT-3′, 5′-GCGAGAGCTTCGACTGCCTCTTCGA-3′, 5′-CCTTAGCGTGAAGATGGCCTCGGGA-3′ and 5′-CGTGCACTTTCAGGCCGGCCGCTTT-3′) from Thermo fisher scientific. Control non-target siRNA (5′-ATCCGCGCGATAGTACGTA-3′) was obtained from Cosmo Bio (Tokyo, Japan). LKO lentivirus constructs expressing shRNA targeting SOCS1 were obtained from Thermo fisher scientific (TRCN0000057063, TRCN0000057064, TRCN0000057065, TRCN0000057066 and TRCN0000057067) and Sigma-Aldrich (TRCN0000378148, TRCN0000378212, TRCN0000356245 and TRCN0000356244). Control LKO vector expressing non-targeting shRNA was obtained from Sigma-Aldrich. For transient knockdown, HEK293T and TE671 cells (3.0×10^5^ cells/well in a 12-well plate) were transfected with 20 pmol of siRNA or 1.0 µg of LKO vector with Lipofectamine 2000 (Thermo Fisher Scientific) or FuGENE6, respectively. Two days post-transfection, total RNA of transfected cells was collected. To establish stable knockdown cells, shRNA was expressed by infection with lentivirus vector pseudotyped with vesicular stomatitis virus G-protein. Infected cells were selected for 10 days in the presence of 4 µg/ml of puromycin. Then, total RNA was prepared and *socs1* mRNA expression level was evaluated by quantitative RT-PCR as described below.

### RNA isolation and quantitative RT-PCR

Total cellular RNA was extracted using RNeasy Mini Kit (QIAGEN Inc., Valencia, CA) according to the manufacturer's instructions. cDNA was prepared from 1.0 µg of total RNA, using oligo(dT)_20_ primer and Superscript III (Thermo fisher scientific). Synthesized cDNA was used as a template for RT-PCR quantification. Quantitative PCR was performed with RT product equivalent to 25 ng of total RNA and specific primer sets for *socs1,* Rh*trim5α* and *gapdh* using SYBR green PCR Kit (Thermo fisher scientific). Primers for quantitative RT-PCR were as follows. *socs1* sense: 5′-GAACTGCTTTTTCGCCCTTA-3′ and *socs1* antisense: 5′-CTCGAAGAGGCAGTCGAAG-3′ [32]. Rh*trim5α* sense: 5′-TTGGATCCTGGGGGTATGTGCTGG-3′ and Rh*trim5α* antisense: 5′-TGATATTGAAGAATGAGACAGTGCAAG-3′ [33], *gapdh* sense: 5′-AAGGTCGGAGTCAACGGATT-3′ and *gapdh* antisense: 5′-CTCCTGGAAGATGGTGATGG-3′ [34]. The mRNA levels were determined by the absolute quantification method using a standard curve or by the ΔΔCT method. RT-PCR was carried out using the StepOne plus quantitative PCR system (Thermo fisher scientific). Results of each mRNA level normalized by *gapdh* mRNA level are shown.

### Immunoprecipitation

HEK293T cells (2.0×10^6^ cells in a 6 cm dish) were co-transfected with 1.0 µg of pRhTRIM5α-HA and 2.0 µg of pHuSOCS1 using FuGENE6. The total amount of plasmids transfected was adjusted to 3.0 µg per sample with pcDNA3.1. Two days after transfection, cells were harvested with 1.0 ml of RIPA buffer. Cell debris were removed by centrifugation. Nonspecifically binding proteins were removed by pre-cleaning with protein G agarose (Thermo fisher scientific) at 4°C for 3 hours. After pre-cleaning, RhTRIM5α and associated proteins were incubated with rat anti-HA antibody and then precipitated with protein G agarose beads. After extensive washing with RIPA buffer, precipitants were resuspended in 15 µl of laemmli sample buffer and subjected to immunoblot analysis.

### VLPs purification

HEK293T cells (2.0×10^6^ cells in a 6 cm dish) were co-transfected with 2.4 µg of proviral plasmid pNL4-3, 2.4 µg of pRhTRIM5α-HA and 2.4 µg of pHuSOCS1 using FuGENE6. The total amount of plasmids transfected was adjusted to 7.2 µg per sample with pcDNA3.1. On the next day of transfection, culture medium was replaced with fresh medium. At 48 hours post-transfection, culture supernatants were harvested through a 0.45 µm syringe filter and subjected to virion purification; virions in filtered supernatants were precipitated through a 20% sucrose cushion by centrifugation (18,700×*g*) at 4°C for 2 hours. Pellets were washed twice with 1×PBS (-), lysed in 10 µl of laemmli sample buffer and subjected to immunoblot analysis.

### Luciferase reporter assay

HEK293T cells (1.5×10^5^ cells/well in a 24-well plate) were co-transfected with 0.05 µg of pGL4.84-EF1α-hRlucCP (EF1α) or pcDNA3.1-luc (CMV) and 0.45 µg of pHuSOCS1 using FuGENE6. The total amount of plasmids transfected was adjusted to 0.5 µg per sample with pcDNA3.1. Two days post-transfection, cells were lysed in 100 µl of lysis buffer and luciferase activity was determined with Dual-Luciferase Reporter Assay System (Promega) as described elsewhere [35].

## Results

### SOCS1 reverses RhTRIM5α-mediated late restriction of HIV-1 replication

**Figure 1:**
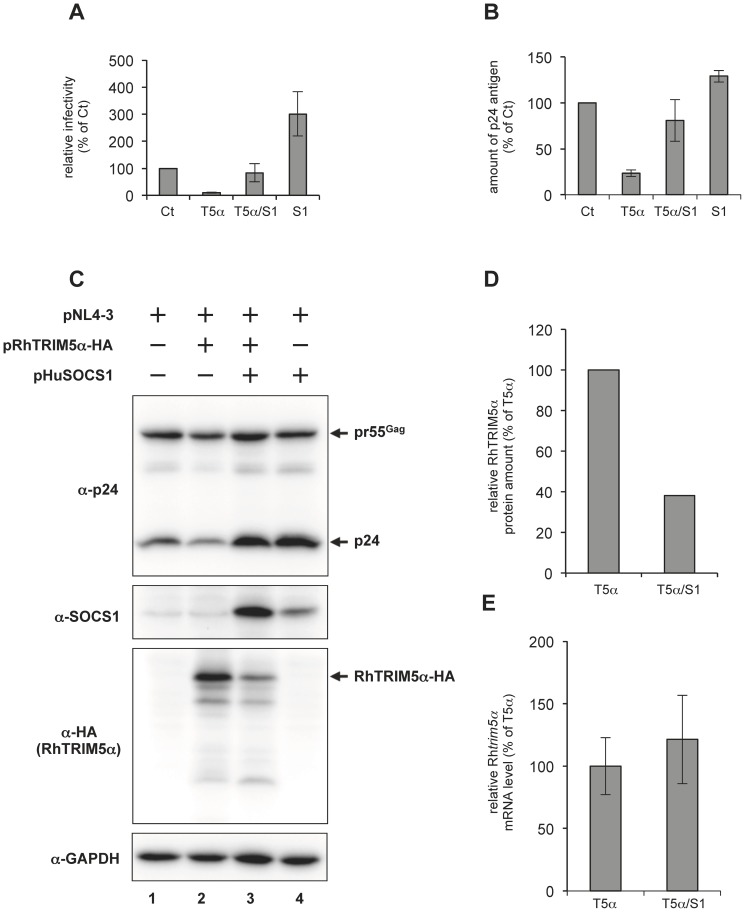
SOCS1 reverses RhTRIM5α-mediated late restriction of HIV-1 replication. (A) HEK293T cells were transfected with 0.1 µg of pNL4-3 and with or without 0.6 µg of pHuSOCS1 (S1) and/or 0.3 µg of pRhTRIM5α-HA (T5α). Ct represents transfection with the control vectors. T5α/S1 indicates that S1 and T5α were co-transfected besides pNL4-3. The total amount of plasmids transfected was adjusted to 1.0 µg per sample with pcDNA3.1. Two days post-transfection, relative viral titer in the supernatants was analyzed with TZM-bl indicator cells. Average of results from four independent experiments is shown with standard deviation. (B) The amount of p24 antigen in the supernatants was quantified with p24-specific ELISA. Data were obtained from the same experimental sets shown in panel A. (C) Twenty-four μg of whole cell lysates were subjected to immnoblot analyses with anti-HIV-1 p24, anti-HA (RhTRIM5α-HA), anti-SOCS1 and anti-GAPDH antibodies. (D) Relative RhTRIM5α protein expression level was determined by densitometry analysis in panel (C). The band intensity for T5α was arbitrarily set as 100%. (E) Relative Rh*trim5α* mRNA expression determined by quantitative RT-PCR. The value for T5α was arbitrarily set as 100%. The results are shown as an average obtained in four independent experiments with standard deviation.

To determine if SOCS1 is functionally involved in RhTRIM5α-mediated late restriction, we examined the effect of SOCS1 over-expression on RhTRIM5α-mediated inhibition of HIV-1 production, where the level of exogenously expressed Rh*trim5α* mRNA was approximately 200-fold higher than that of endogenous *trim5α* mRNA in HEK293T cells. HIV-1_NL4-3_ (NL4-3) was produced in the presence or absence of SOCS1 and/or RhTRIM5α. As shown in figure 1A, NL4-3 titer assayed with TZM-bl indicator cells showed that RhTRIM5α expression reduced infectious HIV-1 production up to 10-fold and that SOCS1 expression slightly enhanced the titer as reported previously [7], [24]. RhTRIM5α expression decreased the amount of p24 released in the supernatant (Figure 1B, compare Ct and T5α) and in producer cells (Figure 1C, compare lanes 1 and 2). In this experimental condition, SOCS1 co-expression elevated the titer of virus released from HEK293T cells that expressed RhTRIM5α to levels comparable to those for control virus (Figure 1A, compare T5α/S1 and Ct). The amounts of p24 antigen in the supernatant (Figure 1B) correlated with infectivity. Immunoblot analysis with anti-p24 antibody revealed that SOCS1 expression elevated the Gag expression level in the presence and absence of RhTRIM5α (Figure 1C). Unexpectedly, SOCS1 reproducibly reduced the expression level of RhTRIM5α (Figure 1, C and D), but did not affect the mRNA level of Rh*trim5α* (Figure 1E). These results indicate that over-expression of SOCS1 reverses RhTRIM5α-mediated late restriction.

We next attempted to examine if endogenous SOCS1 expression influences RhTRIM5α-mediated inhibition of HIV-1 production. We first compared the expression levels of endogenous *socs1* mRNA in HEK293T cells supporting RhTRIM5α's restriction and TE671 cells refractory to it. Quantitative RT-PCR analyses showed that the endogenous expression level of *socs1* in TE671 cells was higher than that in HEK293T cells (Figure S1). Then we attempted to deplete endogenous *socs1* by siRNA transfection, yet no siRNA successfully reduced *socs1* mRNA in HEK293T or TE671 cells. We next hired lentivirus vectors expressing shRNA with puromycin-resistant gene and established over 200 puromycin-resistant cell clones, yet all clones showed *socs1* levels comparable to the control shRNA-transduced clones (data not shown).

### SOCS1 physically interacts with RhTRIM5α

**Figure 2:**
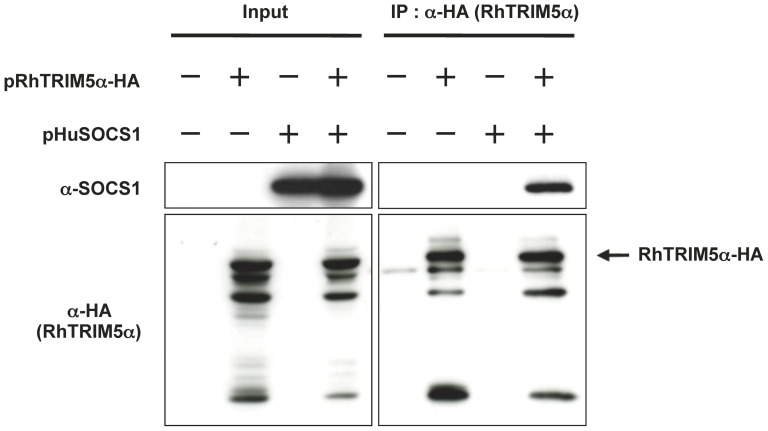
SOCS1 physically interacts with RhTRIM5α. HEK293T cells were co-transfected with 1.0 µg of pRhTRIM5α-HA and 2.0 µg of pHuSOCS1. The total amount of plasmids transfected was adjusted to 3.0 µg per sample with pcDNA3.1. Two days post-transfection, whole cell lysates were subjected to immunoprecipitation for RhTRIM5α with anti-HA antibody. Input lysates (left panels) and precipitated proteins (right panels) were separated by SDS-PAGE and proteins were detected by immunoblot analyses with anti-SOCS1 (upper panels) and anti-HA (RhTRIM5α-HA, lower panels) antibodies.

To know how SOCS1 reverses the late restriction by RhTRIM5α, we first examined if SOCS1 physically interacts with RhTRIM5α by immunoprecipitation (Figure 2). RhTRIM5α was immunoprecipitated from HEK293T cells transfected or not with RhTRIM5α and/or SOCS1. SOCS1 was readily detected in RhTRIM5α precipitates, indicating physical interaction between SOCS1 and RhTRIM5α. In contrast, neither endogenous nor exogenous SOCS1 expressed in TE671 cells was co-immunoprecipitated with RhTRIM5α (data not shown).

**Figure 3:**
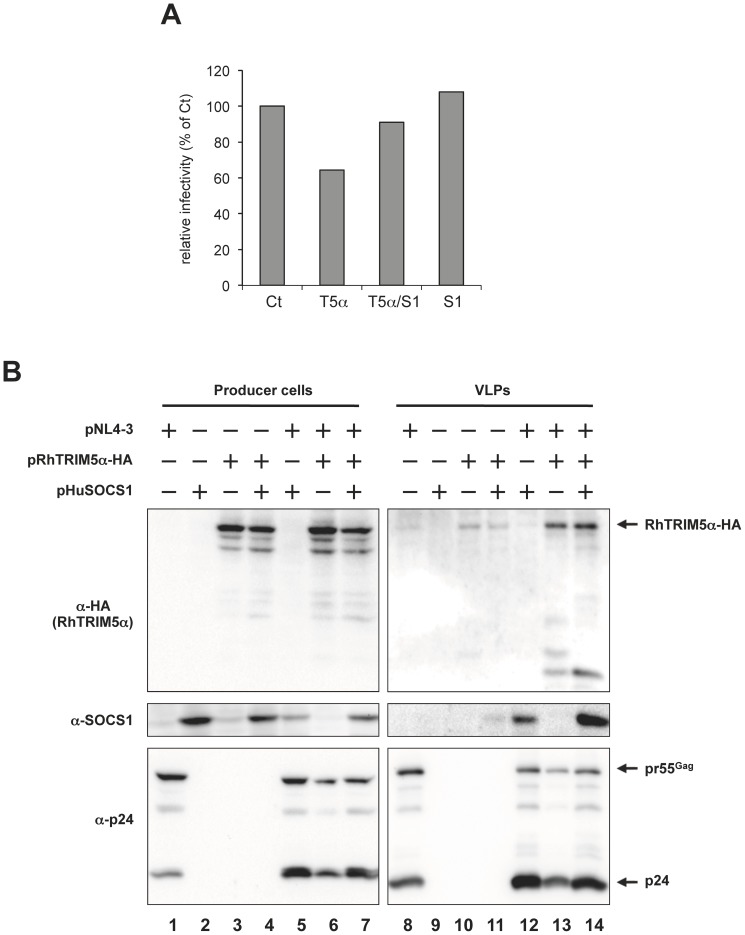
SOCS1 is detected in purified HIV-1 VLPs. HEK293T cells were co-transfected with 2.4 µg of pNL4-3, 2.4 µg of pRhTRIM5α-HA and 2.4 µg of pHuSOCS1. The total amount of plasmids transfected was adjusted to 7.2 µg per sample with pcDNA3.1. (A) Relative viral titer in the supernatants was analyzed with TZM-bl indicator cells two days post-transfection. The titer of virus produced in the absence of RhTRIM5α and SOCS1 (Ct) was arbitrarily set as 100%. S1, T5α or T5α/S1 indicate virions produced in the presence of SOCS1 alone, RhTRIM5α-HA alone or both RhTRIM5α-HA and SOCS1, respectively. Producer cell lysates (B, left panels) and purified HIV-1 VLPs purified through a 20% sucrose layer (B, right panels) were subjected to immunoblot analysis. Proteins were detected with anti-HA (RhTRIM5α-HA, top panels), anti-SOCS1 (middle panels) and anti-HIV-1 p24 (bottom panels) antibodies.

Our previous report showed potential interaction between pr55^Gag^ and RhTRIM5α by forced-incorporation assay [7]. Since SOCS1 was reported to recognize matrix and/or nucleocapsid region of pr55^Gag^ [24], we examined if SOCS1 is incorporated into the HIV-1 VLPs in the presence of RhTRIM5α. To collect detectable amount of VLPs, we employed a modest condition of the late restriction so that about 70% of the control viral production was retained in the presence of RhTRIM5α (Figure 3A). Produced VLPs were purified through a 20% sucrose layer and the VLPs-containing fractions from the same volume of supernatant were subjected to immunoblot analysis (Figure 3B). Both SOCS1 and RhTRIM5α were detected in the purified fraction of VLPs, but barely detectable in the absence of Gag (Figure 3B lanes from 12 to 14). The faint RhTRIM5α bands in the absence of Gag (lanes 10 and 11) may reflect incorporation of a tiny amount of this protein in micro-vesicles. RhTRIM5α expression slightly reduced the amount of Gag proteins in the cell extract and VLPs (compare lanes 6 and 13 to lanes 1 and 8, respectively), while expression of SOCS1 enhanced expression of Gag proteins (compare lanes 5 and 12 to lanes 1 and 8, respectively) as previously reported. When RhTRIM5α and SOCS1 were expressed together, the levels of Gag expression in producer cells and VLPs were comparable to those for cells expressing SOCS1 alone (compare lanes 7 and 14 to lanes 5 and 12, respectively). Moreover, SOCS1 was more abundantly incorporated into purified VLPs released from cells expressing SOCS1 and RhTRIM5α (lane 14) than those expressing SOCS1 alone (lane 12). These results suggest potential interaction of Gag, SOCS1 and RhTRIM5α and raise intriguing questions of where SOCS1 and RhTRIM5α are localized in VLPs and if they keep complexed with Gag, which certainly need further investigation.

### SOCS1 affects RhTRIM5α expression in a dose-dependent manner

**Figure 4:**
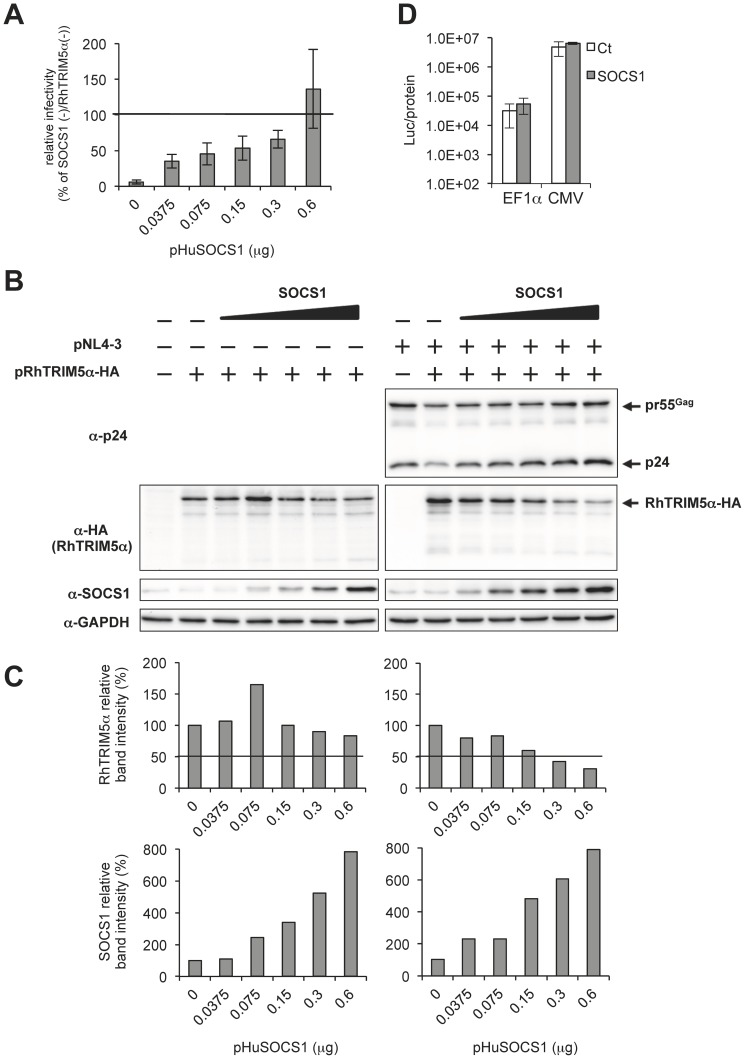
SOCS1 affects RhTRIM5α expression in a dose-dependent manner. HEK293T cells were co-transfected with 0.1 µg of pNL4-3 or pUC18 with increasing amounts of pHuSOCS1 (0, 0.0375, 0.075, 0.15, 0.3 and 0.6 µg) and with or without 0.3 µg of pRhTRIM5α-HA. The amount of plasmid DNA was adjusted to 1.0 µg with pcDNA3.1. Two days post-transfection, viral titer in the culture supernatants was analyzed with TZM-bl indicator cells. (A) Relative viral titer obtained without exogenous SOCS1 and RhTRIM5α was arbitrarily set as 100%. The results are shown as an average of three independent experiments with standard deviation. (B) Whole cell lysates in panel (A) were subjected to immunoblot analyses with anti-HIV-1 p24, anti-HA (RhTRIM5α-HA), anti-SOCS1 and anti-GAPDH antibodies. (C) Relative band intensities of RhTRIM5α and SOCS1 in panel (B) were normalized with that of GAPDH. The normalized band intensity of RhTRIM5α (upper panels) in the absence of SOCS1 and that of SOCS1 (lower panels) without exogenous SOCS1 were arbitrarily set as 100%. (D) Effect of SOCS1 expression on transcriptional activity. HEK293T cell were co-transfected with 0.05 µg of pGL4.84-EF1α-hRlucCP (EF1α) or pcDNA3.1-Luc (CMV) together with 0.45 µg of pcDNA3.1 or pHuSOCS1. The amount of plasmid DNA was adjusted to 0.5 µg with pcDNA3.1. Two days after transfection, luciferase activity was determined and normalized with protein concentration (Luc/protein). The results are shown as an average of three independent experiments with standard deviation.

Immunoblot analyses of cell lysates showed an impaired expression of RhTRIM5α in the presence of SOCS1 (Figure 1D and 3B). Thus, to understand how SOCS1 expression influences RhTRIM5α expression during HIV-1 production, we transfected HEK293T cells with the RhTRIM5α expression plasmid and pNL4-3 or pUC18 together with increasing amounts of the SOCS1 expression plasmid. As shown in figure 4 A and B, viral titer was restored with increasing amounts of SOCS1 in the presence of RhTRIM5α and SOCS1 enhanced the expression of HIV-1 Gag in producer cells in a dose-dependent manner. Additionally, expression of increasing amounts of SOCS1 tended to reduce the amount of RhTRIM5α (Figure 4, B and C, left panels). In the presence of HIV-1, SOCS1 expression impeded RhTRIM5α expression to a greater extent (Figure 4, B and C, right panels). This suggests that Gag somehow enhances destabilization of RhTRIM5α by SOCS1, but its mechanism remains to be studied. To exclude a possibility of SOCS1 generally affecting the transcription/translation, luciferase activity from the CMV and EF1α promoters were analyzed in the presence and absence of SOCS1. The activities of the CMV promoter-driven firefly luciferase and those of the EF1α promoter-driven *Renilla* luciferase were not significantly affected by SOCS1 expression (Figure 4D).

**Figure 5:**
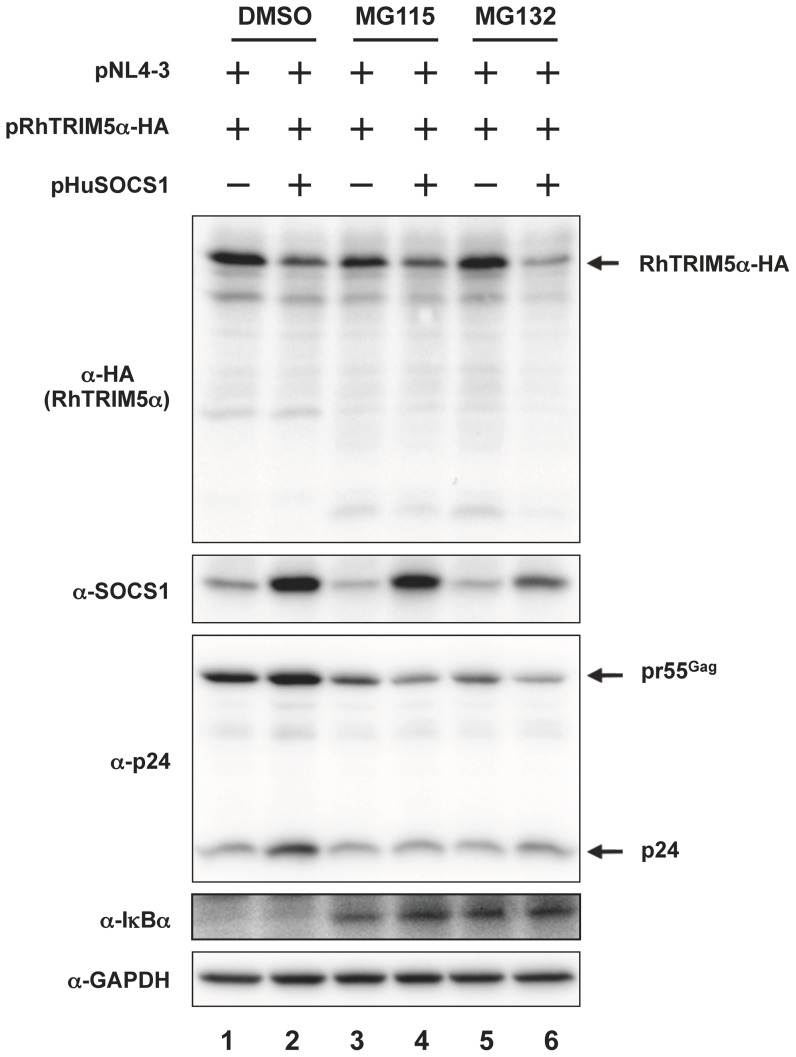
Reduction of RhTRIM5α by SOCS1 is proteasome-independent. HEK293T cells were transfected with 0.1 µg of pNL4-3, 0.3 µg of pRhTRIM5α-HA with or without 0.6 µg of pHuSOCS1. The total amount of plasmids transfected was adjusted to 1.0 µg per sample with pcDNA3.1. Twenty-four hours after transfection, cells were treated with 30 µM of MG115 (lanes 3 and 4) or 30 µM of MG132 (lanes 5 and 6) for 16 hours. Whole cell lysates were subjected to immunoblot analyses with anti-HA (RhTRIM5α-HA, top panel), anti-SOCS1 (middle panel), anti-IκBα and anti-GAPDH (as loading control, bottom panel) antibodies.

It is known that over-expression of SOCS1 induces proteasome-dependent degradation of some proteins including Tel-Jak2 oncogene protein and insulin receptor substrates through the E3 ubiquitin ligase activity dictated by its SOCS box [15], [36]–[41]. Thus, we examined if SOCS1 reduced RhTRIM5α in a ubiquitin-proteasome dependent manner. HEK293T cells that over-expressed RhTRIM5α and SOCS1 were treated with a proteasome inhibitor, MG132 or MG115, for 16 hours and then the expression of RhTRIM5α was analyzed by immunoblotting. The effect of the proteasome inhibitor was verified by the increased expression of IκBα known to be degraded by proteasome [42], [43]. As shown in figure 5, SOCS1 reduced the steady state level of RhTRIM5α without proteasome inhibitor treatment (Figure 5, compare lanes 1 and 2). Treatment with 30 µM of MG115 or 30 µM of MG132 did not restore the expression of RhTRIM5α (Figure 5, compare lanes 3 and 4, 5 and 6, respectively).

## Discussion

In this study we investigated the mechanism of our previous finding, RhTRIM5α-mediated inhibition of HIV-1 production and provided evidence that SOCS1 physically interacts with RhTRIM5α, affects RhTRIM5α expression and facilitates Gag production (Figure 1 and 4). Moreover, both SOCS1 and RhTRIM5α were detected in purified VLPs, suggesting interaction among RhTRIM5α, SOCS1 and pr55^Gag^ (Figure 3). It was reported that SOCS1 recognizes the matrix and nucleocapsid regions in pr55^Gag^ and that SOCS1 rescues pr55^Gag^ from lysosomal degradation in the cytoplasm [24]. Thus, SOCS1 interacts with pr55^ Gag^ in the cytoplasm before viral assembly and this interaction may be stable enough to move SOCS1 with pr55^ Gag^ toward the plasma membrane. The present study showed that RhTRIM5α interacts with SOCS1 in the cytoplasm and that both SOCS1 and RhTRIM5α were detected in purified VLPs. Based on these results, we propose possible models for SOCS1 counteraction to RhTRIM5α-mediated restriction; SOCS1 forms heterodimers with Gag or RhTRIM5α separately to promote degradation of RhTRIM5α; SOCS1 competes with RhTRIM5α for interaction with HIV-1 Gag to increase virus production.

RhTRIM5α-mediated late restriction is a cell-line specific event; HEK293T cells support its antiviral activity, yet TE671 cells do not [8], [9]. In this study, we found that the expression level of endogenous *socs1* in TE671 cells was higher than that in HEK293T cells (Figure S1). Therefore, we attempted to deplete endogenous SOCS1 in producer cells to determine its role in the late restriction, but failed to obtain any SOCS1 knockdown cells. This suggests that endogenous SOCS1 expression was essential for and tightly regulated in TE671 cells. Indeed, it was reported that lentiviral vector-mediated SOCS1 shRNA transduction promoted apoptosis of alveolar epithelial cells [44]. It is also plausible that the siRNA was sensed by intrinsic immunity followed by up-regulation of SOCS1 expression. In fact, in the course of knockdown experiments, control non-targeting siRNA transfection elevated endogenous *socs1* mRNA level (data not shown). Instead of SOCS1 depletion, we observed that neither over-expressed nor endogenous SOCS1 co-immunoprecipitated with RhTRIM5α in TE671 cells, which are refractory to RhTRIM5α-mediated late restriction (data not shown). This suggests that the physical interaction between SOCS1 and RhTRIM5α mediates the late restriction by RhTRIM5α.

Previously, it was reported that HIV-1 Nef induces SOCS1 expression to promote replication by restricting innate immune response [18]. Another report showed that endogenous SOCS1 expression level was elevated in dendritic cells of HIV-1 transgenic rats and peripheral blood mononuclear cells of HIV-1 infected patients [22]. These observations suggested that HIV-1 hires SOCS1 for efficient replication *in vivo*. Rhesus SOCS1 and mouse SOCS1 enhanced HIV-1 production in 293T cells albeit to a lesser extent compared with human SOCS1, while they reversed RhTRIM5α-mediated restriction similarly to human SOCS1 (data not shown). Co-immunoprecipitation analysis indicated that SOCS1 also interacted with HuTRIM5α (data not shown). Interestingly, a recent report from Dr. Luban's laboratory indicated that HuTRIM5α has a role as a pattern reorganization receptor that recognizes retroviral capsid lattice and induces innate immune response through the activation of TAK kinase complex [45]–[47]. It is possible that HIV-1 hires SOCS1 to counteract HuTRIM5α-induced intrinsic immunity.

In conclusion, we have presented evidence indicating that SOCS1 interacts with RhTRIM5α and counteracts RhTRIM5α-mediated restriction of HIV-1 production by reducing functional RhTRIM5α.

## Supporting Information

Figure S1 **Quantitative RT-PCR analysis of endogenous** ***socs1*** **mRNA in HEK293T and TE671 cells.** Endogenous *socs1* mRNA levels in HEK293T and TE671 cells were measured by quantitative RT-PCR with 25 ng of total RNA as a template. The results are shown as an average obtained in four independent experiments with standard deviation.
(PDF)
