# Supplementary material for: Suppressor of Cytokine Signaling 1 Counteracts Rhesus Macaque TRIM5α-Induced Inhibition of Human Immunodeficiency Virus Type-1 Production
Source: PLoS One. 2014 Oct 13;9(10):e109640. doi: 10.1371/journal.pone.0109640 (PMC4195675; doi:10.1371/journal.pone.0109640)
Supplement: Figure S1 — Quantitative RT-PCR analysis of endogenous socs1 mRNA in HEK293T and TE671 cells. Endogenous socs1 mRNA levels in HEK293T and TE671 cells were measured by quantitative RT-PCR with 25 ng of total RNA as a template. The results are shown as an average obtained in four independent experiments with standard deviation. (PDF) [file pone.0109640.s001.pdf]

Supporting information

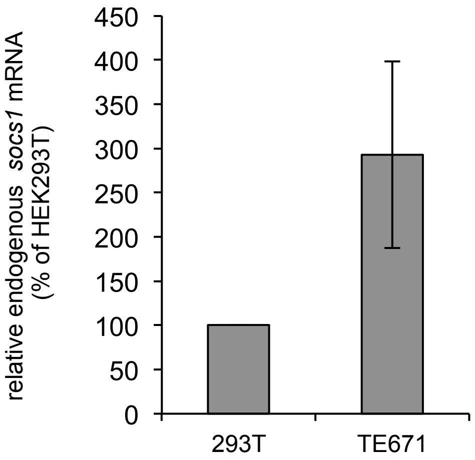

Figure S1. Quantitative RT-PCR analysis of endogenous *socs1* mRNA in HEK293T and TE671 cells. Endogenous *socs1* mRNA levels in HEK293T and TE671 cells were measured by quantitative RT-PCR with 25 ng of total RNA as a template. The results are shown as an average obtained in four independent experiments with standard deviation.
